# Emerging Genomic Trends on Rabies Virus in Davao Region, Philippines, 2018–2021

**DOI:** 10.3390/v15081658

**Published:** 2023-07-30

**Authors:** Jessel Babe G. Capin, Angela Jahn C. Sanque, Maria Noreen J. Eng, Arlene Lagare, Maria Corazon B. Sepulveda, Lyre Anni E. Murao

**Affiliations:** 1 Department of Biological Science and Environmental Studies, College of Science and Mathematics, University of the Philippines Mindanao, Davao City 8000, Philippines; jgcapin@up.edu.ph (J.B.G.C.); acsanque@up.edu.ph (A.J.C.S.); 2 Davao City Veterinarian’s Office, Davao City 8000, Philippines; noreenjasaeng@gmail.com (M.N.J.E.); arlenelagare1967@gmail.com (A.L.); cvo@davaocity.gov.ph (M.C.B.S.)

**Keywords:** canine rabies virus, whole genome sequencing, nanopore technology, biosurveillance, Philippines

## Abstract

Rabies, caused by the rabies virus (RABV), remains a significant public health issue in the Philippines despite efforts to control it. To eliminate rabies by 2030, effective surveillance strategies are crucial. In this study, we examined RABV evolution and phylodynamics in the Davao Region using genome sequences from Davao City and nearby provinces. We adapted the RABV ARTIC Protocol for Oxford Nanopore High-Throughput Sequencing to optimize workflow efficiency under limited resources. Comparing new virus samples collected from June 2019 to June 2021 (*n* = 38) with baseline samples from June 2018 to May 2019 (*n* = 49), new sub-clades were observed in the phylogenetic tree, suggesting divergence from older variants that were previously undetected. Most of the new viruses belonged to the Asian SEA4_A1.1.1 lineage, but new (SEA4_B1 and SEA4_B1.1) and emerging (SEA4_B1.1_E1) lineages that have never been reported in the Philippines were also identified. The baseline study reported phylogeographic clustering of RABV isolates from the same areas. However, this pattern was disrupted in the current biosurveillance, with variants detected in areas outside the original cluster. Furthermore, our findings revealed significant transmission routes between Davao City and neighboring provinces, contrasting with the predominantly intra-city transmission observed in the baseline study. These results underscore the need for ongoing and timely genomic surveillance to monitor genetic diversity changes and the emergence of novel strains, as well as to track alterations in transmission pathways. Implementing cost-effective next-generation sequencing workflows will facilitate the integration of genomic surveillance into rabies control programs, particularly in resource-limited settings. Collaborations between different sectors can empower local laboratories and experts in genomic technologies and analysis.

## 1. Introduction

Rabies is a life-threatening acute encephalomyelitis caused by the rabies virus (RABV), a neurotropic *Lyssavirus* that is primarily transmitted to humans through the bite of domestic dogs [1]. Rabies is an ancient disease, but still remains one of the most neglected diseases, particularly in developing countries. It causes an estimated 60,000 human rabies cases deaths each year; 50% of deaths are in children under the age of 15, mostly in Asia and Africa [2,3]. The World Health Organization is supporting a campaign to eliminate canine-mediated rabies in humans by 2030 [1,4].

The Philippines is a major rabies-endemic country in Southeast Asia, with approximately 200 to 300 human cases of dog-mediated rabies annually. To prevent and control human rabies, Republic Act No. 9482, or the Anti-Rabies Act of 2007, was enacted by the Department of Health in 2007 [5]. The Anti-Rabies Act mandates the local government units to implement the mass vaccination of dogs, control interventions such as impounding and neutering, field control and disposition of unregistered, stray, and unvaccinated dogs, and the conduct of rabies prevention and control information and education campaigns [6]. In Davao City—one of the largest cities in the country with approximately 163,000 dogs—mass vaccination of the canine population was enforced in 2006, while control interventions such as impounding and neutering of free-roaming dogs have been implemented since 2011 [7]. Despite the enhanced implementation of these control measures, the incidence of human rabies in the city has not significantly decreased in recent years [6].

A major challenge in the eradication of the rabies virus is the absence of reliable surveillance data for countries, such as the Philippines where the disease is prevalent. Surveillance is essential for constructing informed infectious disease management and control programs [8]. Genomic data are emerging as an effective surveillance tool through an understanding of pathogen populations, confirming suspected cases, discriminating between strains, identifying infectious agents, and elucidating the evolutionary history and transmission patterns of the virus [9]. Due to breakthroughs in high-throughput sequencing technologies over the last two decades, generating microbial genomes is no longer considered difficult or expensive. Thus, whole-genome sequencing (WGS) has been envisioned as the obvious and inevitable future of diagnostics [10].

In 2014, Oxford Nanopore Technologies (ONT) introduced a portable and affordable sequencing device called the MinION [11,12]. MinION has been used to sequence a wide range of microorganisms from human and other animal clinical samples, which include bacteria, viruses, and fungi [13]. Given its small, agile physical presence and long-read capabilities, the MinION has been used in a number of significant microbiological studies, including the detection of Ebola, Zika virus, and vancomycin-resistant Enterococci [14]. A sequencing workflow for the genomic surveillance of the rabies virus was developed by Brunker et al. [11] which provided genomic data that revealed significant and distinctive insights into rabies spread in Kenya, Tanzania, and the Philippines.

Bacus et al. [15] generated baseline genomic data on rabies cases in Davao City, Philippines, and its neighboring localities between June 2018 to May 2019 using traditional whole genome sequencing via the Sanger method, highlighting the local dynamics of canine rabies. To further monitor the genomic evolution and phylodynamics of the rabies virus in these areas, genomic surveillance was carried out from June 2019 to June 2021 utilizing the Oxford Nanopore MinION sequencing platform. The genetic diversity and evolutionary placement of the new viruses in reference to the baseline data and newly identified transmission routes provide deeper insights into the longer-term impact of rabies interventions in the region, enabling the local government to evaluate and improve on existing strategies.

## 2. Materials and Methods

### 2.1. Sample Collection and Processing

A total of 81 dog brain tissue samples that tested positive for rabies virus in a fluorescent antibody test (FAT) were obtained from the City Veterinarian’s Office (CVO) of Davao City, Philippines from June 2019 to June 2021. Epidemiological data such as location, gender, vaccination status, and the type of ownership of the dogs were recorded. All samples were stored at −80 °C until used. Ethics clearance (IACUC No. 2021-02-004) was obtained from the Institutional Animal Care and Use Committee on 15 July 2021 at Davao Medical School Foundation Inc. (DMSF) and was approved by Dr. Bayani Vandenbroeck, IACUC Chairperson, prior to sample processing. Brain tissue (100–120 mg) was homogenized in a Bead Mill 24 Homogenizer (FisherBrand, Loughborough, UK). The collected supernatant was then used for viral RNA extraction using the Biospin Viral DNA/RNA Extraction Kit (Bioflux, Kolkata, India) following the manufacturer’s protocol. The supernatant from the lysed samples and the RNA extracts were kept at −80 °C until used. RNA extracts were subjected to RT-PCR of the RABV N gene to confirm the identity and the integrity of the RNA extracts [15]. Out of the 81 samples, only 66 were confirmed positive for RABV via RT-PCR.

### 2.2. RABV Genome Enrichment

Viral RNA of the RT-PCR positive samples was reverse transcribed to its complementary DNA (cDNA) using 5X LunaScript RT SuperMix (NEB, New England, Ipswich, MA, USA). This was followed by viral DNA amplification via multiplex PCR using pooled primers [11] and Q5 High-Fidelity Hot-Start DNA Polymerase (NEB). Multiplex PCR primers were prepared based on the RABV Southeast Asia (rabvSEasia) primer scheme, which was produced from Philippine reference sequences: KX148260, KX148263, N-gene 99% consensus sequence of (AB116581, AB116582, AB683592:AB683635), AB981664, KX148255, JN786878, KX148250, KX148254, EU293111, KX148248, and KX148266 [11]. The following PCR cycling conditions were initially applied: heat inactivation at 98 °C for 30 s, 30–40 cycles of denaturation at 98 °C for 15 s, and a combined extension and annealing step at 65 °C for 5 min. For the next batches of samples, touchdown PCR was performed, wherein the temperature during the combined extension and annealing step was decreased from 65 °C by 0.1 °C every cycle for the first 25 cycles and then kept at 62.5 °C. PCR products were purified using the NEBNext Sample Purification Bead (NEB), and concentrations were measured using a Qubit dsDNA High Sensitivity assay kit on a Qubit 3.0 fluorometer (ThermoFisher, Waltham, MA, USA). A negative control included at the cDNA synthesis stage was used to check for contamination. If the concentration of the negative control was above 1 ng/μL, sample preparation was repeated from RNA extraction.

### 2.3. Library Preparation

Library preparation for the MinION was conducted using Ligation Sequencing 1D and Native Barcoding kits (Oxford Nanopore Technologies, Oxford, UK) on FLO-MIN106 flow cells according to the manufacturer’s instructions. RABV DNA was first end-repaired and dA-tailed using an end-prep master mix followed by the ligation of barcodes from the ONT Native Barcoding kit. Samples were pooled into a single tube and purified with SPRI beads (NEB). Adapter ligation of the barcoded amplicon pool was performed using the NEBNext ARTIC Companion Kit according to the manufacturer’s instructions, and the sample was cleaned up using SPRI beads (NEB). The acceptable amount of genetic material in the final library for all runs was between 5–50 fmol [11] as measured by a Qubit dsDNA High Sensitivity assay kit (Thermofisher) on a Qubit 3.0 fluorometer (Thermofisher).

### 2.4. Nanopore Sequencing

Sequencing was carried out on a DELL Inspiron 3511 computer with a 1920 × 1080 Intel Core i7-1165G processor, 8 GB DDR4 3200 MHz, and 512 GB SSD storage using MinKnow version 20.10.6 (ONT, Oxford, UK) with live basecalling turned off. The ligation sequencing and native barcoding kits used during library preparation were specified in the MinKnow interface. Run length varied between 16 to 18 h depending on the amount of sequence data generated over time. All other settings were set to the default value. After sequencing, all liquid in the flow cell was removed from the waste channel through the waste port. About 400 μL of wash mix containing wash mix and wash diluent ONT EXP-WSH004 (ONT, Oxford, UK) was loaded into the priming port. The mixture was then removed after 30 min. After that, storage buffer was slowly added through the priming port to allow the storage of the flow cell for subsequent library additions.

### 2.5. Bioinformatics Data Processing and Analysis

Consensus sequences were generated from the raw reads using the RABV version of the ARTIC network open-source bioinformatics pipeline [16]. The resulting sequences were then aligned with the Southeast Asia reference sequence (KX148260) using MAFFT version 7 [17]. The identity and coding region coverage of the sequences were determined using the online RABV-GLUE genotyping tool (http://rabv-glue.cvr.gla.ac.uk (accessed on 8 July 2022)), while the whole genome coverage for each sample was manually calculated by dividing the total number of non-N bases by the total length of the sequence × 100%. SAMtools version 1.16.1 was then used to calculate the mean read depth and breadth of coverage of the sequences using the BAM files generated from the RABV ARTIC pipeline [18]. Sequences for phylogenetic analysis were selected based on the following criteria: whole genome coverage (where depth at each position > 20X) is greater than 70% [19], and the breadth of coverage (where depth at each position > 1X) obtained via SAMtools is greater than 90% [11]. Bammix version 1.0.0 was used to search for nucleotide mixtures among samples suspected to be contaminated during the sequencing run using filters on base quality (q > 7), mapping quality (m > 40), depth of coverage (>20X), base proportion (>25%), and read position (30–11,620 bp) [20].

Phylogenetic inference was performed using the discrete continuous-time Markov chain model (CTMC) via BEAST version 1.10.4. A total of 38 new viruses and 49 samples from the baseline study (Accession No.: OQ326736 to OQ326784) were included in the analysis. Genomic data of reference sequences from the Philippines (KM148260), Brazil (KM594034, KM594033, KM594041, and KM594028), and China (JN234411) was used as the outgroup in constructing the phylogenetic tree. All sequences were first aligned using MUSCLE in MEGA version 11 [21], where the resulting multiple sequence alignment was trimmed and manually cleaned by removing all gaps. The best-fit model with the least Akaike Information Criterion (AIC) was also identified using MEGA. An executable xml file was prepared using BEAUTi version 1.10.4 with the GTR + G + I DNA substitution and site heterogeneity model, a length of 200 million, uncorrelated relaxed molecular clock, and coalescent constant size model. Ancestral state reconstruction was enabled using an asymmetric trait substitution model. The rest of the tree priors were set to the default value. The resulting log files from BEAST were then assessed using Tracer version 1.7.2 [22], ensuring that the estimated sampling size (ESS) values for most of the continuous parameters are sufficient (>200). TreeAnnotator version 1.10.4 was then used to construct a maximum clade credibility (MCC) tree, where the first 20 million states were specified as burn-in, and the posterior probability limit was set to 0.0. FigTree version 1.4.4 was used to visualize the resulting phylogenetic tree.

NextStrain was also used to generate a TimeTree phylogeny of the sampled sequences [23], while RABV lineage assignment was performed using the tool Method for Assignment, Definition and Designation of Global Lineages (MADDOG) [24]. Phylogeographic analysis was conducted using the Bayesian stochastic search variable selection (BSSVS) approach in BEAST software with the following criteria: strong support (30 > BF > 10), very strong support (100 > BF > 30), and decisive (BF > 100). The results were then visualized through SPREAD3 version 0.9.6 [25].

## 3. Results

### 3.1. Sample Characteristics

**Figure 1:**
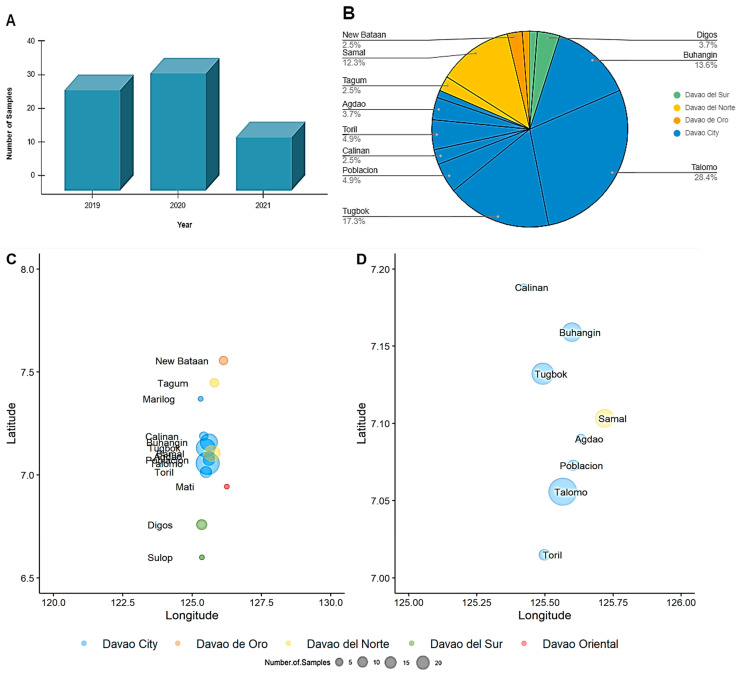
Distribution of RABV-positive samples in Davao City and neighboring provinces in Davao Region, Philippines. (**A**) Collection period of samples from June 2019 to June 2021. (**B**) Municipalities and provinces of the collected samples. (**C**) Relative geographical location of provinces and corresponding number of samples collected during the study period. (**D**) Magnified view of (**C**) for sampling locations within Davao City and Samal Island.

A total of 81 reported samples that tested positive for rabies virus in a fluorescent antibody test (FAT) conducted by the Davao CVO were processed in this study. Out of 81, 30 samples were obtained from June to December 2019, 35 in the year 2020, and 16 from January to June 2021 (Figure 1A). The Davao Region is located in Southern Mindanao of the Philippines and is composed of one major city, Davao City, and five provinces Davao de Oro, Davao del Norte, Davao del Sur, Davao Oriental, and Davao Occidental. The samples were collected from reported cases of rabies in various locations of Davao City (76.54%) and the neighboring provinces of Davao del Sur (4.94%), Davao del Norte (14.81%), and Davao de Oro (3.70%) (Figure 1B–D).

Out of the 81 FAT-positive brain samples, 66 (81.5%) were confirmed by RABV N RT-PCR and subjected to RABV whole genome enrichment via multiplex PCR. Modifications of the RABV MinION sequencing protocol established by Brunker et al. [11] were adopted in this study (Table 1). The RNA extraction kit and native barcoding kits were changed to accommodate available resources. Other modifications were introduced to improve the sequencing workflow, which was first tested out in a trial run. For the multiplex PCR, nucleic acid concentrations of the initial batch of purified amplicons were too low to be detected. Touchdown PCR was subsequently employed to increase primer specificity, in which the reaction temperature was decreased from 65 °C by 0.1 °C until it reached 62.5 °C, resulting in purified amplicons with concentrations ranging from 1.39 to 108 ng/μL. The oldest samples from 2019 (*n* = 9) were excluded from library preparation due to undetectable nucleic acid concentrations post-PCR cleanup.

About 5 ng of input DNA was initially used for end-prep and barcode ligation reactions during the trial run of the library preparation (Table 1). The barcoded samples were then pooled together, totaling to 30 ng of genetic material for adapter ligation. Prior to adapter ligation, the pooled amplicons were purified, resulting in a concentration of only 0.7 ng/μL. In subsequent runs, the input DNA was increased from 5 ng to 50 ng. To minimize DNA loss during cleanup, short fragment buffer (SFB) and 80% ethanol were used for the bead washings. Precise incubation temperatures were also applied by using a thermocycler instead of a heat block. With these modifications, the nucleic acid concentration of the barcoded amplicon pool in four batches was recorded to be 0.84 to 3.78 ng/μL (Table 2). A total of 57 samples (86.4% of sample input) were subjected to nanopore sequencing.

During sequencing, the run was extended to 16 h or more with the basecalling mode off instead of 6 h with the basecalling mode on to improve the read number (Table 1). To maximize resources, flow cells were recycled for a total of two uses. In batches sequenced consecutively using the same flow cell, traces of contamination from the previous run were observed despite the flow cell wash step. For example, thousands of reads were recorded from the negative control of the second batch, enough to create a consensus sequence with >70% coverage despite having a nucleic acid concentration of <1 ng/μL prior to pooling during library preparation. The negative control in question shares the same native barcode with a RABV-positive sample from the previous run. To investigate possible contamination in this batch, the binary alignment map (BAM) files of the samples ran in the reused flow cell were further examined for nucleotide mixtures using bammix. Samples (*n* = 11) that overlapped barcodes with the previous batch were flagged to have possible nucleotide mixtures in multiple positions. Thus, these sequences were discarded and not used in downstream analysis. It is important to note, however, that nucleotide mixtures were also observed in clean samples albeit only a few hundred reads. Given that unique barcodes and flow cells were used when running them, clean sequences were still included in the analysis.

### 3.2. RABV Whole Genome Sequences

Consensus sequences were generated for 46 rabies viruses (80.7% of sequenced samples). One sample was not confirmed to be RABV through RABV-GLUE due to very low sequence read depth, while the remaining 45 samples were identified to belong to the Asian SEA4 clade (Table A1). However, only 38 out of the 45 viruses had acceptable whole genome coverage and breadth of coverage based on the set threshold values. A few samples (*n* = 7) that dated back to 2019 had very low coverage and were not used for downstream phylogenetic and phylodynamic analysis (Table A1).

**Figure 2:**
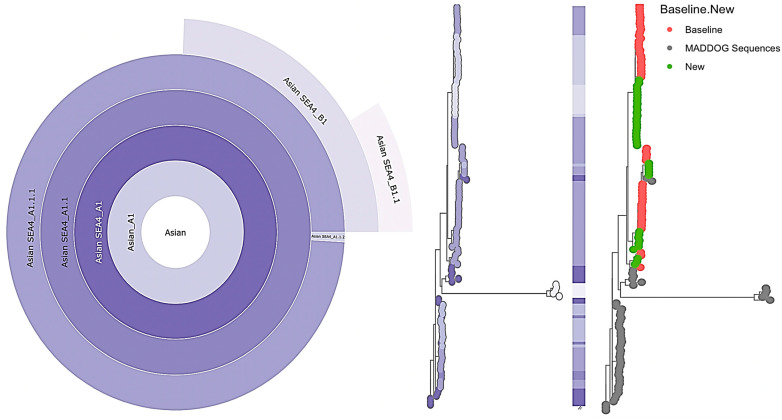
Lineage assignments of rabies whole genome sequences from Davao City, Philippines against the MADDOG Global Reference Set, 2018–2021. Hierarchical relationships of new and existing lineages are represented in a sunburst plot in the leftmost panel, expanding outward from major (Asian_A1) to minor lineages (Asian SEA4_B1.1). The middle panel shows a phylogenetic tree of the input sequences (*n* = 87) plus all sequences from relevant lineages, with tips colored according to the color scheme from the sunburst plot. Sequences from the baseline study (*n* = 49) are indicated in the rightmost panel in red circles, new sequences from this study (*n* = 38) are in green, while sequences from relevant existing lineages in the MADDOG repository are in gray (Table A3).

Lineage designation for the sampled viruses, including those from the baseline study in 2018–2019 [15], was based on the global reference set in the MADDOG database (Table A2). The nomenclature system by Rambaut et al. [26] was applied. The majority of the sequences from the Davao Region were assigned to the lineage Asian SEA4_A1.1.1 (69.4% of the old sequences and 63.2% of the new sequences), which emerged from the SEA4_A1.1 parent lineage (Figure 2). Some of the remaining sequences were suspected to belong to two new lineages descending from the A1.1.1 lineage: SEA4_B1 (30.6% of the old sequences and 7.9% of the new sequences) and SEA4_B1.1 (26.3% of the new sequences), while one (2.6%) of the new sequences was assigned to the SEA4_A1.1.2 lineage. Note that the A1.1.2 and B1.1 lineages were identified only among the new samples. Interestingly, one potentially emerging but currently undersampled lineage (Asian SEA4_B1.1_E1) was also identified by MADDOG among the new viruses.

### 3.3. Genetic Diversity and Phylogeographic Distribution of RABV

**Figure 3:**
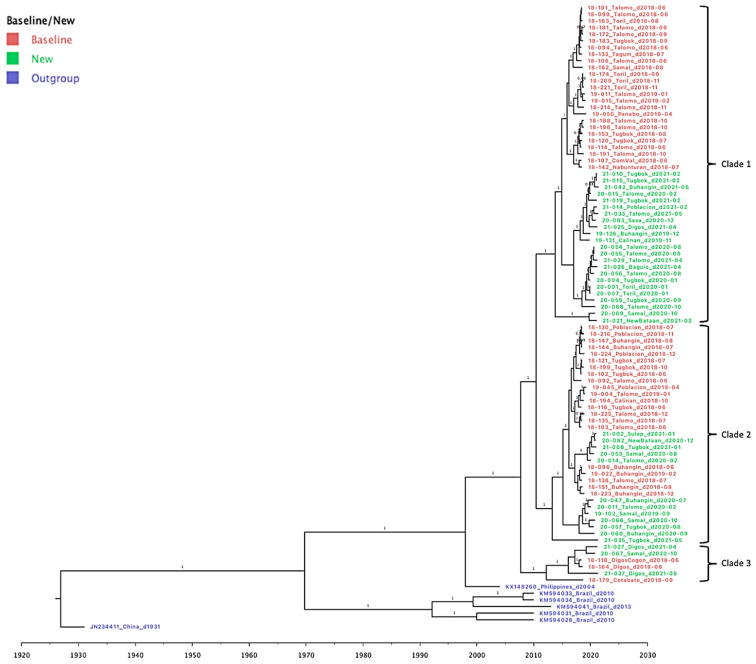
Bayesian phylogeny of 87 reported RABV samples from Davao City and from neighboring provinces in Davao Region, Philippines. Samples are labeled according to the Sample ID_ District/Province_Barangay_Date of sampling. Genomic data of reference sequences from the Philippines (KM148260), Brazil (KM594034, KM594033, KM594041, and KM594028), and China (JN234411) were used as the outgroup in constructing the phylogenetic tree. Major clades are indicated in brackets, while old and new samples are in red and green text, respectively.

Phylogenetic analysis was performed to assess the genetic relationships of the rabies viral strains circulating within the different districts in Davao City and its neighboring provinces in the Davao Region. Samples from the baseline study in 2018–2019 [15] were included to monitor changes in virus clades and transmission. A phylogenetic tree was produced through Bayesian inference, mapping a total of 87 reported samples from June 2018 to June 2021 (Figure 3). Three major clades were observed, with each composed of samples from both baseline (highlighted in red) and new sequence data (highlighted in green). In all three clades, the newly sampled viruses from 2019–2021 formed a divergent sub-clade with respect to the baseline sequences from 2018–2019 with ancestral placement in the phylogenetic tree. The most recent common ancestor for each clade dated back to 2014, 2013, and 2010 for Clades 1, 2, and 3, respectively. Bayesian posterior probabilities (≥0.95) are indicated on the branch labels.

**Figure 4:**
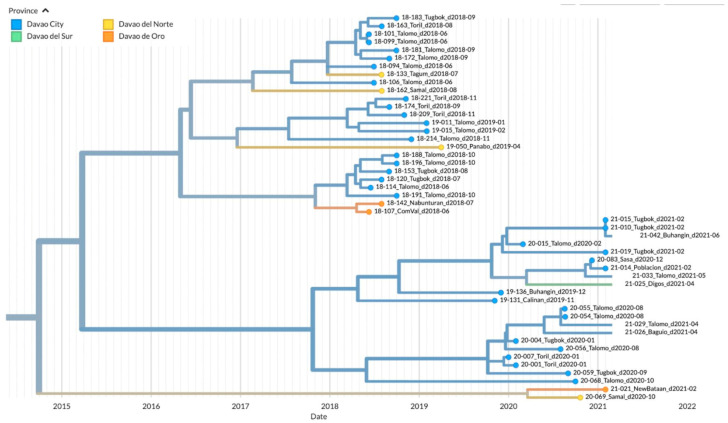
TreeTime phylogeny of reported samples from Davao City and neighboring provinces from Davao Region, Philippines (Clade 1). Samples are labeled according to the Sample ID_ District/Province_Barangay_Date of sampling and color-coded according to the city or province.

To visualize the geographic distribution of the samples among the provinces in the Davao Region, a time-resolved phylogenetic tree was inferred. Similar to the Bayesian inferred phylogeny, three major clades were evident. Clade 1 was composed of 40 samples from Davao City: Toril (*n* = 6), Tugbok (*n* = 8), Talomo (*n* = 20), Calinan (*n* = 1), Baguio (*n* = 1), Buhangin (*n* = 2), Sasa (*n* = 1), and Poblacion (*n* = 1); 4 samples from Davao del Norte: Tagum (*n* = 1), Panabo (*n* = 1), and Samal (*n* = 2); 3 samples from Davao de Oro: New Bataan (*n* = 1) and Compostela Valley (*n* = 2); and 1 sample from Digos, Davao del Sur (Figure 4). The inferred date of divergence of this clade from its parent clade was 30 September 2014, indicating that the most recent common ancestor for these samples dated back to 2014. The time resolution of the samples was consistent with the sampling dates.

**Figure 5:**
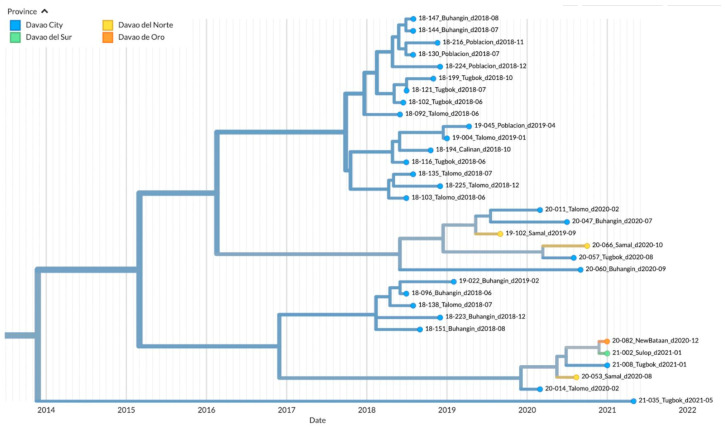
TreeTime phylogeny of reported samples from Davao City and neighboring provinces from Davao Region, Philippines (Clade 2). Samples are labeled according to the Sample ID_ District/Province_Barangay_Date of sampling and color-coded according to the city or province.

The majority of the samples mapped in Clade 2 were from the various districts of Davao City: Buhangin (*n* = 8), Poblacion (*n* = 4), Tugbok (*n* = 7), Talomo (*n* = 8), and Calinan (*n* = 1) (Figure 5). Newly sampled viruses from neighboring provinces such as Sulop (*n* = 1) of Davao del Sur, Samal (*n* = 3) of Davao del Norte, and New Bataan (*n* = 1) of Davao de Oro were also found in this clade. The most recent common ancestor for all samples in Clade 2 dated back to 2014, with the time resolution of the samples consistent with the sampling dates.

**Figure 6:**
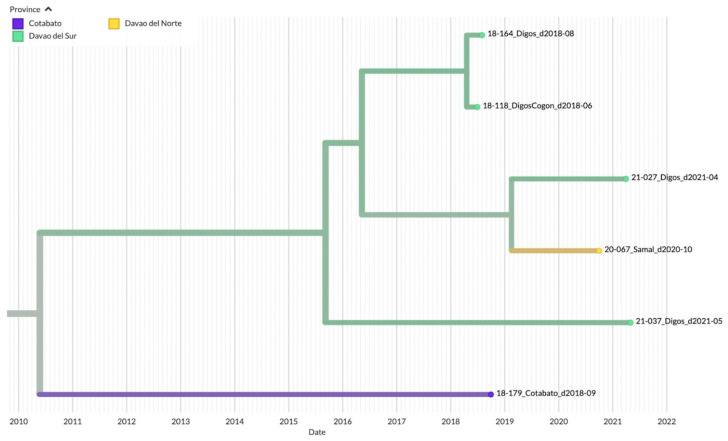
TreeTime phylogeny of reported samples from Davao City and neighboring provinces from Davao Region, Philippines (Clade 3). Samples are labeled according to the Sample ID_ District/Province_Barangay_Date of sampling and color-coded according to the city or province.

Clade 3 on the other hand was mainly composed of samples from Davao del Sur in the southern part of the Davao Region (*n* = 4), although samples from the Northern Davao del Norte (Samal, *n* = 1) and outside Davao Region (Cotabato City, *n* = 1) were also mapped (Figure 6). The inferred date of divergence of Davao Region samples from the Cotabato strain was 25 May 2010, indicating that the most common ancestor for these samples dated back to 2010. The time resolution of the samples was also consistent with the sampling dates.

### 3.4. Phylodynamics of RABV in Davao Region

**Figure 7:**
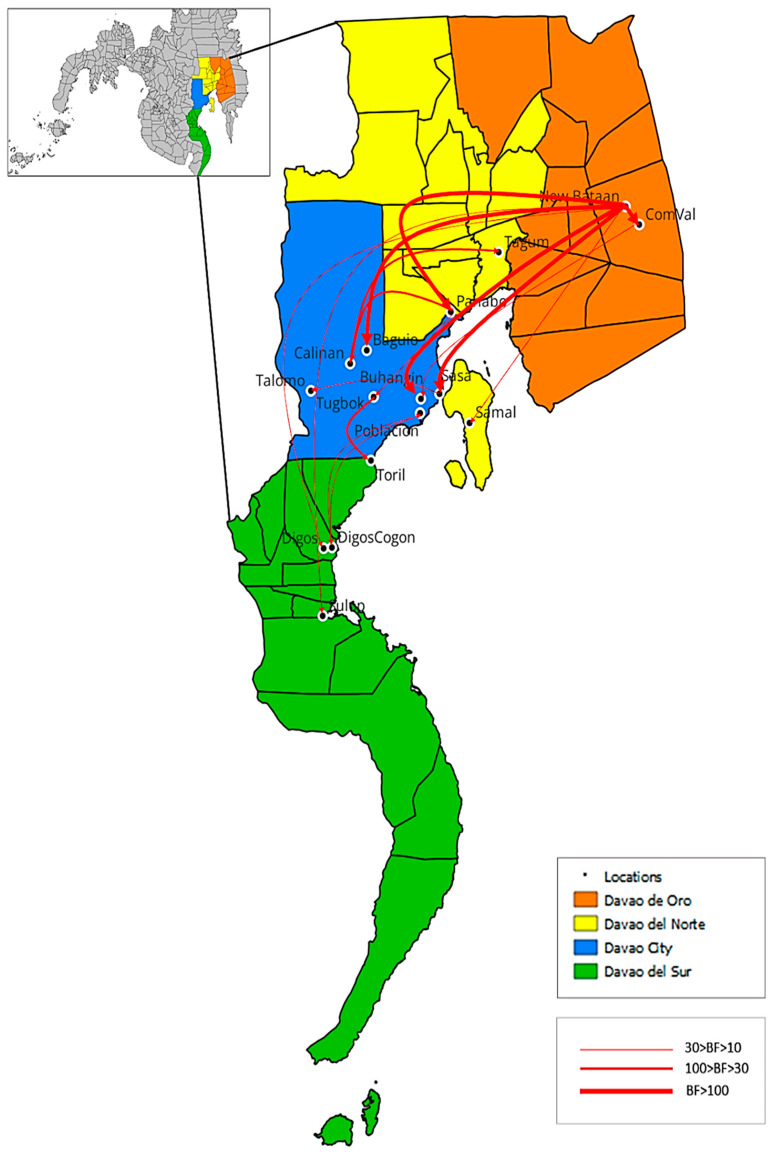
Transmission routes of reported RABV in Davao Region. Sixteen significant transmission routes with Bayes factor (BF) support > 10 were identified. Strong support: 30 > BF > 10; very strong support: 100 > BF > 30; decisive: BF > 100. Map Source: QGIS version 3.22.10.

The ancestral state of the phylogeny from the BEAST analysis was reconstructed to shed light on the transmission routes of rabies in the Davao Region. A total of 16 routes were identified with strong to decisive Bayes factor support (BF > 10) as shown in Table 3 and Figure 7. Out of the sixteen routes, two were recorded as intra-city transmission within Davao City, nine inter-city transmissions between Davao City and neighboring provinces, one intra-province transmission within a Davao Region province, and four inter-province transmissions. The reconstructed transmission routes are specific only for the RABV isolates captured in this study and from Bacus et al. [15] and do not cover other potentially circulating variants during the study period.

## 4. Discussion

### 4.1. Emerging Trends on RABV Diversity and Transmission Patterns

We obtained the whole genome sequence of 38 RABV isolates from Davao City, Philippines collected between June 2019 and June 2021 using NGS by Oxford Nanopore Technology. Integrating our new sequences to the baseline data of 49 RABV isolates in Davao City collected from 2018 to 2019 [15] showed that the more recent viruses collected between 2019 to 2021 diverged from 2018 to 2019 older variants, suggesting the emergence of new sub-clades. This hypothesis is supported by the lineage analysis via MADDOG, where 28.9% of the 2019–2021 sequences belonged to new sub-lineages dubbed SEA4_A1.1.2 and SEA4_B1.1; these were previously not detected among isolates in the baseline study for the Davao Region. Instead, the isolates in the baseline study were all composed of the SEA4 B1 lineage. The A1.1.2 lineage was first detected in Luzon in 2013 [11], while the B1 and B1.1 lineages from the Davao Region detected in 2018–2021 have never been reported elsewhere in the country. Note that the A1.1.2 and B1.1 lineages were detected among the new but not the baseline samples, suggesting they emerged in Davao Region sometime after 2019. This finding is consistent with our TreeTime phylogenetic data. However, it is also possible that these variants were just not captured in the baseline study, hence the prior unavailability of sequences in these lineages.

We also report an emerging but currently undersampled lineage, SEA4_B1.1_E1. New lineages are assigned only when at least ten samples are identified under it within a five-year period [24]. Only five samples were associated with the B1.1_E1 lineage, all of which were isolated in the Davao Region, Philippines between 2019–2021. Hence, continuous monitoring is recommended. To our knowledge, we are the first to report the B1.1_E1 lineage in the country, although it is difficult to say at this point whether this is emerging only in the Davao Region or more broadly in the Philippines.

Mapping our data with the baseline sequences also revealed that the recent samples formed new sub-clades which diverged from much older ancestral strains, instead of branching out from the posterior lineages. This implies the possible existence of at least one undetected, unidentified, older variant circulating around Davao City. New and previously undetected variants could have advanced characteristics on transmissibility, virulence, and vaccine resistance, among others. Thus, the epidemiological fitness of these new sub-clades and whether they would outcompete the other clades in the Davao Region will be of great interest.

Aside from the emergence of new lineages and clades, notable changes in transmission pathways were also observed. In the baseline surveillance from 2018 to 2019, RABV variants were clustered according to geographic locations [15]. Our genomic surveillance in the subsequent years from 2019–2021 revealed that variants previously associated with a geographic area have been introduced to other locations. For example, Clade 1 was mainly composed of samples from Davao City and neighboring areas in the northern parts of the Davao Region [15]. However, our analysis showed that these variants have now reached Digos City in Davao del Sur. Clade 2 on the other hand was initially exclusively found in Davao City but is now populated with new viruses from other provinces, such as Davao del Norte, Davao de Oro, and Davao del Sur.

These observations were validated by the phylogeographic spread analysis where transmission across districts and provinces was evident. Compared to the predominantly inter-city transmission in the baseline study [15], most of the movements in the current study were between Davao City and its neighboring provinces, suggesting heightened long-distance transmissions. Davao City is a major urban hub located at the center of Davao Region in Southern Mindanao [27]. The interconnecting factors between human socioeconomic and population density might determine the transmission dynamics of RABV [28]. Thus, frequent rural-to-urban viral transmission within the Davao Region is highly possible. However, the noted changes may also be due to a widened range of sample submissions from areas outside Davao City (approximately 25% of total sample collection in the current study as opposed to 16% in the baseline study of Bacus et al., 2021).

A similar scenario was observed in our analysis of the Island Garden City of Samal in Davao del Norte. All three clades contained samples from Samal, suggesting that various areas of the Davao Region are involved in the transmission events for this island. Although bodies of water can be a strong barrier to rabies transmission [29], Samal Island is a popular tourist destination in Southern Mindanao and is only a few kilometers away from the mainland. The high level of human movement from several parts of the Davao Region to Samal Island, therefore, makes the island highly susceptible to human-mediated inter-island transmission. Human-mediated inter-island transmission has been previously reported in other regions in the Philippines [29] and in other countries such as Thailand [28] and North Africa [30].

The long-distance spread of RABV is likely due to human-mediated displacement of dogs infected with rabies, a significant driver in repeated and successful reintroduction of RABV in rabies-free areas [30]. Multiple studies have reported that frequent reintroductions counteract local rabies elimination after vaccination campaigns, allowing rabies to continually spread in endemic areas like the Philippines [31,32,33]. Long-distance transmission has also been observed among recent RABV variants in Ghana, which belonged to both Africa 1a and Africa 1b sub-lineages, contrary to earlier reports that a single lineage (Africa 2) circulates in West Africa [34]. Africa 1a has been previously known to dominate in Northern and Eastern Africa, while Africa 1b has been detected in Eastern, Central, and Southern Africa, suggesting the probable repeated introduction of RABV from other parts of Africa into Ghana [30,35]. With the long-distance and inter-island transmission events observed in Davao City and Samal, local government units (LGU) should explore interventions that will strictly regulate such movements to limit transmission and prevent the introduction or re-introduction of the virus. For example, Samal was about to be declared rabies-free in 2011 (personal communication, Davao City Veterinary Office), but our current findings suggest that weak border control may have forestalled this.

Our analysis also showed that the Talomo District in Davao City had the highest number of reported cases, making it the city’s RABV hotspot. This supports findings from the baseline study [15], in which this district has been identified as both donor and recipient in the RABV transmission chain within Davao City. Population structure is a driving force for rabies maintenance, particularly in urban areas where human and dog population densities are high [31]. As a result, the emergence and recurrence of rabies cases in large urban areas are caused by an increasing human and dog population [36,37].

### 4.2. Genomic Surveillance in a Low Resource Setting

Our new findings on formerly undetected and emerging lineages and new transmission pathways highlight the importance of continued rabies monitoring through genomic surveillance. Unlike traditional clinical reporting, genomic surveillance can provide deeper insights into virus characteristics and behavior that could be crucial in guiding the rabies endgame [9]. Expanding surveillance including samples outside the target locality could capture long distance transmission pathways that would answer key questions about viral circulation, and potential host shifts, and provide insights into persisting lineages [38].

In many third-world countries where rabies remains to be endemic, funding opportunities for next-generation sequencing are limited [39]. Hence, available resources have to be maximized and an efficient workflow for genomic surveillance is very important. Relative to the laborious and time-consuming conventional sequencing that was utilized in the baseline study [15], the MinION workflow provided a speedy yet affordable platform for WGS. In our study, 86% of genome-enriched samples made it to sequencing. Whole genome data were successfully generated for 67% of these samples after MinION workflow optimization, where output was improved by increasing input DNA, utilizing alternative techniques such as touchdown PCR, and optimizing clean-up steps.

The use of reagents other than the prescribed kits in the sequencing workflow developed by Brunker et al. [11] may have led to variable efficiency. This could explain why only 81% of the reported RABV-positive samples tested positive in our targeted RT-PCR confirmatory step after viral RNA extraction. The age of the samples and storage conditions have to be considered as well. In total, 15 samples repeatedly tested negative during targeted RT-PCR, most of which (73%) were shelved for more than two years prior to the conduct of the study. Among those that were sequenced, the whole genome coverage for older samples (14%) was recorded to be significantly lower than the threshold value of 90% [11]. This suggests that the quality of the tissue samples and the viral RNA itself might have been compromised during storage. Loss of genetic material during long periods of storage or during transport could be reduced by keeping samples in an appropriate preservation buffer [40]. Alternatively, sequencing samples immediately after collection might increase the percent yield.

Reusing flow cells should be conducted cautiously. We found that the ONT storage buffer can clog up waste ports when not properly and completely removed from the waste channel as the residual buffer can later crystalize inside the flow cell. Moreover, we found that reusing flow cells can result in a low read number (i.e., fewer Fast5 files after a 16 h runtime) when the remaining pore number after the first run is less than 400. We also note contamination in the reused flow cell, where native barcodes from the preceding run appeared during the demultiplexing step, even when the flow cell was incubated with the ONT wash reagents for 30 min after the first use. This was supported by our analysis using the bammix tool, which revealed traces of nucleotide mixtures in multiple positions of the samples in question. Surprisingly, nucleotide mixtures were also detected even in clean samples run in unique flow cells (<1% of the total genome). This may indicate possible lapses in the reading efficiency and accuracy of the nanopores in the R9.4.1 flow cell or may be due to errors during basecalling given that the “fast” mode was selected in this study rather than the “high accuracy” option. It is important to consider, however, that the bammix tool counts possible nucleotide mixtures using BAM files as input. The medaka tool (https://github.com/nanoporetech/medaka (accessed on 8 December 2022)) in the RABV ARTIC pipeline was used in this study for generating the consensus sequences from the same BAM files, which guarantees that the true variant is called regardless of any basecalling or nanopore errors, so long as there is enough coverage at each position. Hence, clean samples (i.e., samples processed in unique R9.4.1 flow cells and using unique barcodes) were processed for subsequent phylogenetic analysis given that they pass all criteria set in this study and that the negative control processed along with the samples had concentration < 1 ng/μL prior to library preparation. We recommend extending incubation time with the DNase-containing wash buffer for an hour or more to fully eliminate contaminating samples. We also suggest using different sets of barcodes for consecutive runs in the same flow cell, if possible, to avoid barcode overlapping.

The introduction of efficient and less expensive NGS workflows will be a strong impetus for the inclusion of genomic surveillance in rabies control programs, especially in low-resource settings. This comes with the need to also empower local laboratories that will be capable of implementing the technology with expertise in genomic analysis. This is achievable through the collaboration of academia and public health and veterinary agencies.

## 5. Conclusions

Our study reports a follow-up on the genomic surveillance of the rabies virus in the Southern Philippines. Here, we shifted from conventional sequencing to next-generation sequencing using the Oxford Nanopore High-Throughput Sequencing Technology, which allowed us to efficiently and economically process samples in bulk and in a shorter period of time. The modifications introduced in our sequencing workflow validated the utility of the technology in low-resource settings and may be adopted in other laboratories. Our findings revealed emerging trends in the genetic diversity and transmission patterns of RABV in the Davao Region, highlighting the value of consistent monitoring through genomic surveillance. The inclusion of genomic surveillance in rabies control programs may be facilitated by efficient and less expensive next-generation sequencing workflows, especially in low-resource settings. This would necessitate capacity building of local laboratories and assistance from genomic experts, which can be realized through inter-sectoral collaborations.

## Figures and Tables

**Table 1:** Modifications in the RABV MinION sequencing workflow of Brunker et al. (2020).

Procedure	Challenges Encountered	Original Protocol	Modification
RNA Extraction	Prescribed kit is unavailable	Zymo Research Quick-RNA miniprep kit, 2 mL ceramic tubes (1.4 mm), and Terralyzer	Biospin Viral DNA/RNA Extraction Kit, 2 mL ceramic tubes (2.8 mm), Bead Mill Homogenizer
Multiplex PCR	Low concentration of PCR products	Conventional multiplex PCR	Touchdown multiplex PCR
Library Preparation	Low concentration post barcode ligation	5 ng DNA input per sample; 70% ethanol for cleanup wash step	50 ng DNA input per sample; Freshly prepared 80% ethanol for cleanup wash step
	Ultra II Ligation Module for Native Barcoding not available in purchased kit	Ultra II Ligation Master Mix and Ligation Enhancer	Blunt/TA Ligase Master Mix diluted with NFW
Sequencing	Low read number Recycling of flow cells	Run can finish in six hours, basecalling mode on for fast basecalling n/a	Run extended to 16 h or more, basecalling mode off Yes, but excluded sequences that overlapped barcodes with samples from previous run

**Table 2:** Total nucleic acid concentration of the pooled amplicons and libraries.

Sequence Batch No.	No. of Samples ^a^	Concentration of Barcoded Amplicon Pool (ng/μL)	Concentration of Final Library (ng/μL)
1	12 ^b^	3.78	0.956
2	17	1.39	1.28
3	17	1.18	1.21
4	17	0.84	0.14

^a^ Negative control not included in count; ^b^ Samples were processed in duplicate.

**Table 3:** Bayes factor-supported transmission routes of reported rabies viruses within Davao Region from 2018–2021.

From	To	Support
**Intra-city transmission**		
Sasa ^1^	Talomo ^1^	Strong
Tugbok ^1^	Toril ^1^	Very strong
**Inter-city transmission**		
Poblacion ^1^	DigosCogon ^2^	Strong
ComVal ^4^	Buhangin ^1^	Strong
DigosCogon ^2^	Poblacion ^1^	Strong
Panabo ^3^	Tugbok ^1^	Strong
Calinan ^1^	Tagum ^3^	Very strong
Panabo ^3^	Calinan ^1^	Very strong
New Bataan ^4^	Baguio ^1^	Decisive
New Bataan ^4^	Sasa ^1^	Decisive
New Bataan ^4^	Buhangin ^1^	Decisive
**Intra-province transmission**		
New Bataan ^4^	ComVal ^4^	Decisive
**Inter-province transmission**		
New Bataan ^4^	Digos ^2^	Strong
New Bataan ^4^	Sulop ^2^	Strong
New Bataan ^4^	Samal ^3^	Strong
New Bataan ^4^	Panabo ^3^	Decisive

^1^ Sample collected from Davao City; ^2^ Sample collected from Davao del Sur; ^3^ Sample collected from Davao del Norte; ^4^ Sample collected from Davao de Oro.

## Data Availability

Data are contained within the article or appendices. All other data that support the findings of this study are available from the corresponding authors upon reasonable request.

## Appendix A

**Table A1:** Rabies whole genome sequence profile using RABV-GLUE.

Sequence Batch No.	Sample ID	Identified as RABV?	Closest Full Genome Ref Seq	Whole Genome Coverage	Breadth of Coverage	Coding Region Coverage				
						N	P	M	G	L
1	27-21	Yes	KX149259	94%	98%	100%	100%	100%	100%	91.2%
	29-21	Yes	KX149259	96%	99%	100%	100%	100%	100%	94.4%
	33-21	Yes	KX149259	97%	99%	100%	100%	100%	100%	97.5%
	35-21	Yes	KX149259	92%	99%	100%	100%	100%	100%	90.9%
	37-21	Yes	KX149259	97%	99%	100%	100%	100%	100%	97.5%
	42-21	Yes	KX149259	97%	99%	100%	100%	100%	100%	97.5%
2	47-20	Yes	KX149259	84%	92%	100%	100%	100%	100%	80.2%
	53-20	Yes	KX149259	79%	94%	100%	100%	100%	100%	80.2%
	54-20	Yes	KX149259	91%	97%	100%	100%	100%	100%	90.9%
	55-20	Yes	KX149259	92%	96%	100%	100%	80.1%	100%	90.9%
	56-20	Yes	KX149259	94%	96%	100%	100%	100%	100%	94.5%
	57-20	Yes	KX149259	86%	91%	100%	100%	100%	100%	83.8%
	59-20	Yes	KX149259	93%	96%	100%	100%	100%	100%	94.5%
	60-20	Yes	KX149259	87%	94%	100%	100%	100%	100%	80.3%
	66-20	Yes	KX149259	90%	97%	100%	100%	100%	100%	83.8%
	67-20	Yes	KX149259	97%	98%	100%	100%	100%	100%	97.5%
	68-20	Yes	KX149259	96%	98%	100%	100%	80.1%	87.1%	94.3%
	69-20	Yes	KX149259	92%	98%	100%	100%	100%	100%	94.5%
	82-20	Yes	KX149259	88%	99%	100%	100%	100%	100%	83.4%
	83-20	Yes	KX149259	94%	94%	100%	100%	100%	100%	87.4%
	02-21	Yes	KX149259	87%	96%	100%	100%	100%	100%	83.4%
	08-21	Yes	KX149259	88%	98%	100%	100%	100%	100%	83.4%
	10-21	Yes	KX149259	95%	99%	100%	100%	100%	100%	94.5%
3	14-21	Yes	KX149259	97%	99%	100%	99.9%	100%	99.9%	97.5%
	15-21	Yes	KX149259	94%	98%	100%	99.9%	100%	99.9%	94.5%
	19-21	Yes	KX149259	96%	99%	100%	99.9%	100%	99.9%	94.5%
	21-21	Yes	KX149259	96%	99%	99.9%	99.9%	100%	99.9%	94.5%
	25-21	Yes	KX149259	94%	98%	100%	99.9%	100%	99.9%	94.5%
	26-21	Yes	KX149259	94%	97%	100%	99.9%	100%	99.9%	90.9%
4	98-19	Yes ^b^	KX149259	38%	50%	15.6%	75.7%	63.1%	41.3%	35.5%
	101-19	Yes ^b^	KX149259	25%	33%	15.6%	75.7%	31.2%	17.0%	21.6%
	105-19	Yes ^b^	KX149259	25%	32%	15.6%	75.7%	31.2%	17.0%	21.6%
	118-19	Yes ^b^	KX149259	32%	51%	0.0%	71.3%	31.2%	41.3%	35.5%
	122-19	Yes ^b^	KX149259	47%	56%	15.6%	75.7%	63.1%	60.8%	49.9%
	131-19	Yes	KX149259	82%	94%	78.3%	100%	80.1%	100%	80.6%
	102-19	Yes	KX149259	85%	93%	83.8%	100%	100%	100%	83.4%
	107-19	Yes ^b^	KX149259	69%	89%	63.3%	100%	80.1%	69.8%	63.7%
	135-19	Yes ^b^	KX149259	80%	89%	83.9%	100%	100%	100%	72.3%
	136-19	Yes	KX149259	90%	96%	83.2%	100%	100%	100%	90.9%
	01-20	Yes	KX149259	88%	94%	83.9%	100%	100%	100%	84.9%
	04-20	Yes	KX149259	92%	96%	84.3%	100%	100%	100%	91.0%
	07-20	Yes	KX149259	95%	98%	98.9%	100%	100%	100%	94.5%
	08-20 ^a^	No ^a^	NA ^a^	NA ^a^	2%	0.0%	0.0%	0.0%	0.0%	0.0%
	11-20	Yes	KX149259	80%	90%	83.9%	100%	100%	100%	77.7%
	14-20	Yes	KX149259	88%	93%	100%	100%	100%	100%	83.4%
	15-20	Yes	KX149259	95%	96%	98.9%	100%	100%	100%	94.5%

^a^ Very low sequence read depth, excluded from downstream analysis; ^b^ Whole genome coverage and breadth coverage below threshold, excluded from downstream analysis.

**Table A2:** MADDOG reference sequence.

Sample ID	Country	Year	Lineage
AB573762	Japan	2006	Asian SEA4
AB573763	Japan	2006	Asian SEA4
EU086201	Philippines	1994	Asian SEA4
EU086202	Philippines	1994	Asian SEA4
EU086203	Philippines	2000	Asian SEA4
EU086204	Philippines	2001	Asian SEA4
KF620487	Taiwan	2012	Asian SEA5
KF620488	Taiwan	2012	Asian SEA5
KF620489	Taiwan	2013	Asian SEA5
KP860149	Taiwan	2013	Asian SEA5
KP860153	Taiwan	2013	Asian SEA5
KX148259	Philippines	1994	Asian SEA4
KX148260	Philippines	2004	Asian SEA4
KX148261	Philippines	1994	Asian SEA4
KX148262	Philippines	1994	Asian SEA4
KX148263	Philippines	1994	Asian SEA4
LC571945	Japan	2006	Asian SEA4
LC571946	Japan	2020	Asian SEA4
MN726836	Philippines	2013	Asian SEA4
MN726837	Philippines	2013	Asian SEA4
MN726838	Philippines	2013	Asian SEA4
MN726839	Philippines	2015	Asian SEA4
MN726840	Philippines	2015	Asian SEA4
MN726841	Philippines	2014	Asian SEA4
MN726843	Philippines	2012	Asian SEA4
MN726846	Philippines	2015	Asian SEA4
MN726847	Philippines	2017	Asian SEA4
MN726848	Philippines	2016	Asian SEA4
MN726851	Philippines	2014	Asian SEA4
MN726853	Philippines	2012	Asian SEA4
MN726854	Philippines	2015	Asian SEA4
MN726855	Philippines	2016	Asian SEA4
MN726856	Philippines	2012	Asian SEA4
MN726858	Philippines	2016	Asian SEA4
MN726861	Philippines	2015	Asian SEA4
MN726862	Philippines	2013	Asian SEA4
MN726865	Philippines	2014	Asian SEA4
MN726866	Philippines	2014	Asian SEA4
MN726867	Philippines	2012	Asian SEA4
MN726868	Philippines	2016	Asian SEA4
MN726869	Philippines	2013	Asian SEA4
MN726870	Philippines	2014	Asian SEA4
MN726871	Philippines	2013	Asian SEA4
MN726872	Philippines	2017	Asian SEA4
MN726873	Philippines	2014	Asian SEA4
MN726874	Philippines	2012	Asian SEA4
MN726879	Philippines	2012	Asian SEA4
MN726881	Philippines	2015	Asian SEA4
MN726882	Philippines	2019	Asian SEA4
MN857169	Philippines	2019	Asian SEA4

**Table A3:** Lineage designation of new and baseline samples.

Sample ID	New/Baseline	Length	Year	Lineage
21-002_Sulop_d2021-01	New	10,370	2021	Asian SEA4_A1.1.2
21-008_Tugbok_d2021-01	New	10,551	2021	Asian SEA4_A1.1.1
21-010_Tugbok_d2021-02	New	11,331	2021	Asian SEA4_B1.1
20-047_Buhangin_d2020-07	New	9965	2020	Asian SEA4_A1.1.1
20-053_Samal_d2020-08	New	9481	2020	Asian SEA4_A1.1.1
20-054_Talomo_d2020-08	New	10,851	2020	Asian SEA4_A1.1.1
20-055_Talomo_d2020-08	New	11,035	2020	Asian SEA4_A1.1.1
20-056_Talomo_d2020-08	New	11,195	2020	Asian SEA4_A1.1.1
20-057_Tugbok_d2020-08	New	10,219	2020	Asian SEA4_A1.1.1
20-059_Tugbok_d2020-09	New	11,039	2020	Asian SEA4_A1.1.1
20-060_Buhangin_d2020-09	New	10,347	2020	Asian SEA4_A1.1.1
20-066_Samal_d2020-10	New	10,737	2020	Asian SEA4_A1.1.1
20-067_Samal_d2020-10	New	11,626	2020	Asian SEA4_A1.1.1
20-068_Talomo_d2020-10	New	11,397	2020	Asian SEA4_A1.1.1
20-069_Samal_d2020-10	New	11,013	2020	Asian SEA4_B1
20-082_NewBataan_d2020-12	New	10,550	2020	Asian SEA4_A1.1.1
20-083_Sasa_d2020-12	New	10,473	2020	Asian SEA4_B1.1
21-027_Digos_d2021-04	New	11,221	2021	Asian SEA4_A1.1.1
21-029_Talomo_d2021-04	New	11,422	2021	Asian SEA4_A1.1.1
21-033_Talomo_d2021-05	New	11,619	2021	Asian SEA4_B1.1
21-035_Tugbok_d2021-05	New	10,958	2021	Asian SEA4_A1.1.1
21-037_Digos_d2021-05	New	11,570	2021	Asian SEA4_A1.1.1
21-042_Buhangin_d2021-06	New	11,587	2021	Asian SEA4_B1.1
18-092_Talomo_d2018-06	New	11,800	2018	Asian SEA4_A1.1.1
18-094_Talomo_d2018-06	New	11,800	2018	Asian SEA4_A1.1.1
18-096_Buhangin_d2018-06	New	11,800	2018	Asian SEA4_A1.1.1
18-099_Talomo_d2018-06	New	11,800	2018	Asian SEA4_A1.1.1
18-101_Talomo_d2018-06	New	11,800	2018	Asian SEA4_A1.1.1
18-102_Tugbok_d2018-06	New	11,800	2018	Asian SEA4_A1.1.1
18-103_Talomo_d2018-06	New	11,800	2018	Asian SEA4_A1.1.1
18-106_Talomo_d2018-06	New	11,800	2018	Asian SEA4_A1.1.1
18-107_ComVal_d2018-06	New	11,800	2018	Asian SEA4_B1
18-114_Talomo_d2018-06	New	11,800	2018	Asian SEA4_B1
18-116_Tugbok_d2018-06	New	11,800	2018	Asian SEA4_A1.1.1
18-118_DigosCogon_d2018-06	New	11,800	2018	Asian SEA4_A1.1.1
18-120_Tugbok_d2018-07	New	11,800	2018	Asian SEA4_B1
18-121_Tugbok_d2018-07	New	11,800	2018	Asian SEA4_A1.1.1
18-130_Poblacion_d2018-07	New	11,800	2018	Asian SEA4_A1.1.1
18-133_Tagum_d2018-07	Baseline	11,800	2018	Asian SEA4_A1.1.1
18-135_Talomo_d2018-07	Baseline	11,800	2018	Asian SEA4_A1.1.1
18-138_Talomo_d2018-07	Baseline	11,800	2018	Asian SEA4_A1.1.1
18-142_Nabunturan_d2018-07	Baseline	11,800	2018	Asian SEA4_B1
18-144_Buhangin_d2018-07	Baseline	11,800	2018	Asian SEA4_A1.1.1
18-147_Buhangin_d2018-08	Baseline	11,800	2018	Asian SEA4_A1.1.1
18-151_Buhangin_d2018-08	Baseline	11,800	2018	Asian SEA4_A1.1.1
18-153_Tugbok_d2018-08	Baseline	11,800	2018	Asian SEA4_B1
18-162_Samal_d2018-08	Baseline	11,800	2018	Asian SEA4_A1.1.1
18-163_Toril_d2018-08	Baseline	11,800	2018	Asian SEA4_A1.1.1
18-164_Digos_d2018-08	Baseline	11,800	2018	Asian SEA4_A1.1.1
18-172_Talomo_d2018-09	Baseline	11,800	2018	Asian SEA4_A1.1.1
18-174_Toril_d2018-09	Baseline	11,800	2018	Asian SEA4_B1
18-179_Cotabato_d2018-09	Baseline	11,800	2018	Asian SEA4_A1.1.1
18-181_Talomo_d2018-09	Baseline	11,800	2018	Asian SEA4_A1.1.1
18-183_Tugbok_d2018-09	Baseline	11,800	2018	Asian SEA4_A1.1.1
18-188_Talomo_d2018-10	Baseline	11,800	2018	Asian SEA4_B1
18-191_Talomo_d2018-10	Baseline	11,800	2018	Asian SEA4_B1
18-194_Calinan_d2018-10	Baseline	11,800	2018	Asian SEA4_A1.1.1
18-196_Talomo_d2018-10	Baseline	11,800	2018	Asian SEA4_B1
18-199_Tugbok_d2018-10	Baseline	11,800	2018	Asian SEA4_A1.1.1
18-209_Toril_d2018-11	Baseline	11,800	2018	Asian SEA4_B1
18-214_Talomo_d2018-11	Baseline	11,800	2018	Asian SEA4_B1
18-216_Poblacion_d2018-11	Baseline	11,800	2018	Asian SEA4_A1.1.1
18-221_Toril_d2018-11	Baseline	11,800	2018	Asian SEA4_B1
18-223_Buhangin_d2018-12	Baseline	11,800	2018	Asian SEA4_A1.1.1
18-224_Poblacion_d2018-12	Baseline	11,800	2018	Asian SEA4_A1.1.1
18-225_Talomo_d2018-12	Baseline	11,800	2018	Asian SEA4_A1.1.1
19-004_Talomo_d2019-01	Baseline	11,800	2019	Asian SEA4_A1.1.1
19-011_Talomo_d2019-01	Baseline	11,800	2019	Asian SEA4_B1
19-015_Talomo_d2019-02	Baseline	11,800	2019	Asian SEA4_B1
19-022_Buhangin_d2019-02	Baseline	11,800	2019	Asian SEA4_A1.1.1
19-045_Poblacion_d2019-04	Baseline	11,800	2019	Asian SEA4_A1.1.1
19-050_Panabo_d2019-04	Baseline	11,800	2019	Asian SEA4_B1
19-102_Samal_d2019-09	Baseline	10,122	2019	Asian SEA4_A1.1.1
19-131_Calinan_d2019-11	Baseline	9804	2019	Asian SEA4_B1
19-136_Buhangin_d2019-12	Baseline	10,697	2019	Asian SEA4_B1.1
20-001_Toril_d2020-01	Baseline	10,531	2020	Asian SEA4_A1.1.1
20-004_Tugbok_d2020-01	Baseline	10,958	2020	Asian SEA4_A1.1.1
20-007_Toril_d2020-01	Baseline	11,284	2020	Asian SEA4_A1.1.1
20-011_Talomo_d2020-02	Baseline	9553	2020	Asian SEA4_A1.1.1
20-014_Talomo_d2020-02	Baseline	10,535	2020	Asian SEA4_A1.1.1
20-015_Talomo_d2020-02	Baseline	11,378	2020	Asian SEA4_B1.1
21-014_Poblacion_d2021-02	Baseline	11,547	2021	Asian SEA4_B1.1
21-015_Tugbok_d2021-02	Baseline	11,182	2021	Asian SEA4_B1.1
21-019_Tugbok_d2021-02	Baseline	11,404	2021	Asian SEA4_B1.1
21-021_NewBataan_d2021-02	Baseline	11,411	2021	Asian SEA4_B1
21-025_Digos_d2021-04	Baseline	11,243	2021	Asian SEA4_B1.1
21-026_Baguio_d2021-04	Baseline	11,163	2021	Asian SEA4_A1.1.1
